# Over-expression of human cystatin C in pterygium *versus* healthy conjunctiva

**DOI:** 10.1186/1471-2415-13-6

**Published:** 2013-02-27

**Authors:** Luis Fernando Barba-Gallardo, Javier Ventura-Juárez, David Kershenobich Stalnikowitz, Rafael Gutiérrez-Campos, Eugenia Torres-Bernal, Luis Fernando Torres-Bernal

**Affiliations:** 1 Optometry Department, Center for Health Sciences, Autonomous University of Aguascalientes, Aguascalientes, Mexico; 2 Morphology Department, Center for Basic Sciences, Autonomous University of Aguascalientes, Aguascalientes, Mexico; 3 Governing Board UNAM, National Autonomous University of Mexico, Mexico City, Mexico; 4 Chemistry Department, Center for Basic Sciences, Autonomous University of Aguascalientes, Aguascalientes, Mexico; 5 INOVA Vision Institute, Aguascalientes, Mexico; 6 Medicine Department, Center for Health Sciences, Autonomous University of Aguascalientes, Aguascalientes, Mexico

**Keywords:** Pterygium, Human cystatin C, Proliferation

## Abstract

**Background:**

A prospective, non-randomised, transversal and comparative study, carried out in INOVA Vision Institute and Autonomous University of Aguascalientes. Pterygium is an important illness that affects 22% people from tropic and equatorial zones. Is an inflammatory process caused by UV rays, and it has a behavior similar to a neoplasm. For this study was taken into consideration 191 samples from the INOVA Vision Institute, Aguascalientes, Mexico. Include 73 pterygia samples, which were obtained during resection under sterile conditions. 44 normal conjunctiva samples were obtained from the same patients when harvesting the conjunctival autograft, or from other patients undergoing extracapsular cataract extraction from the superior bulbar region. Tears from patients with pterygium (n = 50) and normal volunteers (n = 24) were obtained using a calibrated glass micro capillary tube. The surgical conjunctiva and pterygia samples were subjected to reverse-transcription polymerase chain reaction (RT-PCR), western blot, and immunohistochemistry. Tears were analyzed by enzyme-linked immunosorbent assays.

**Methods:**

This was a prospective, non-randomised study involving 191 biological samples taken from patients with pterygium and normal volunteers, whom were operated under local anaesthesia by either complete resection of the lesion with primary closure, or resection with conjunctival autograft. Tissue samples were fixed in 10% formaldehyde. Sections were routinely stained with hematoxylin and eosin. HCC expression was evaluated by reverse-transcription polymerase chain reaction (RT-PCR), immunohistochemistry, and by western blotting. All tears samples were analyzed by enzyme-linked immunosorbent assays (ELISA).

**Results:**

Expression levels and distribution patterns of HCC in normal conjunctiva and pterygium. Higher levels of HCC mRNAs and proteins were detected in pterygium compared with a normal conjunctiva. Immunohistochemistry revealed that HCC was localized in the apical cells of the epithelium in the normal conjunctiva. In contrast, HCC was detected in all extension of epithelial tissue, from apical to basal cells in pterygia. The concentration of HCC protein in tears was higher in patients with pterygium versus controls.

**Conclusion:**

HCC may play an important role in protecting normal conjunctiva, and regulating inflammatory conditions of the anterior ocular surface.

## Background

A pterygium is a fibrovascular lesion of the ocular surface that can display aggressive clinical behavior and occasionally threaten vision [1]. The lesion consists of an initial disruption of the corneal-conjunctival barrier, characterized by extensive cell proliferation, inflammation and connective tissue remodeling, from the limbus to the central cornea [2]. Epidemiological data support that chronic exposure to UV light has a prominent role in the pathogenesis of pterygium that affect 22% people from tropic and equatorial zones [3]. Girolamo et al., established the role of matrix metalloproteinases (MMPs) and their inhibitors in the degradation of extracellular matrix (ECM) components (proteoglycans, glycoproteins and collagen, I and III) in pterygium. They suggested that inhibitors of MMP-1 and MMP-3 have a protective role against ECM degradation [4]. Defects in biological mechanisms controlling protease activities can have numerous effects, such as neurodegeneration, cardiovascular diseases, osteoporosis, arthritis, and metastases of cancers [5]. These pathological entities are activated by chronic inflammation, angiogenesis and tissue proliferation, all of which occur in pterygium. Cystatins have been identified as proteins with a particular sequence motif that form equimolar, tight and reversible bonds with cysteine peptidase. These cystatins represent a group of potent, non-covalent, competitive inhibitors of mammalian lysosomal cysteine proteinases that have been conserved throughout evolution. Cystatins inhibit cysteine proteinases belonging to the C1 superfamily, including plant papain and the mammalian cathepsins B, C, H, K, L, and S [6]. An important member of this protein family is human cystatin C (HCC), a non-glycosylated and low molecular weight (14 kDa) protein that is present in almost all human fluids, including tears [7]. HCC has not been studied extensively in ophthalmic pathologies. Paraoan et al., discovered a possible role for cystatin in the neuroepithelium during macular degeneration in human eyes; they also studied other structures of the eye (iris, ciliary body, lens epithelium and ciliary body epithelium) using immunohistochemical methods [8]. Another study showed that the level of cystatin C in tears made up 10% of the total protein content [9]. To date, there are no studies regarding the presence of cystatin C in the pterygium of human eyes. The objective of our study was to determine the expression levels of HCC in patients with pterygium.

## Methods

This was a prospective, non-randomised study involving 191 biological samples taken from patients with pterygium and normal volunteers. HCC expression was evaluated using several techniques. Written informed consent was obtained for each patient. Biological samples were taken during surgery (pterygia and conjunctivas) or consultation (tears) from the anterior segment department of INOVA Vision Institute, Aguascalientes, Mexico. All experiments were carried out in accordance with the Helsinki Declaration and approved by the medical ethics committee of the INOVA Vision Institute. 52 surgical of primary pterygia samples from persons its age ranging between 55 to 65 years old (median age 55.21 ± 10 years), the 21 recurrent pterygia samples were obtained from persons which ages ranging between 58 to 63 years old (median age 65.12 ± 14 years old), the samples were obtained during resection under sterile conditions. 44 normal conjunctiva samples from persons which ages ranging between 52 to 68 years old (median age 62.57 ±6 years) the conjunctivas were obtained from the same patients when conjunctival autografts were harvested, or from other patients undergoing extracapsular cataract extraction from the superior bulbar region. Tears from 50 patients with pterygium and 24 normal volunteers who ages ranging between 51 to 67 years old (median age 61.31 ±9 years) were obtained using a calibrated glass micro capillary tube. One-third of tissue samples (pterygia and conjunctivas) were analyzed by reverse-transcription polymerase chain reaction (RT-PCR), one-third by immunohistochemistry, and the remainder by western blotting. All tears samples were analyzed by enzyme-linked immunosorbent assays (ELISA).

### Surgical approach

Patients with pterygium were operated under local anaesthesia by either complete resection of the lesion with primary closure, or resection with conjunctival autograft [10]. The pterygium head was removed, using a sharp blade, from the anterior corneal surface. The remainder of the body tissue was dissected in a subconjunctival plane. All pterygia were taken from the nasal region, except for one pterygium of temporal origin. Normal conjunctiva samples were taken from the superior bulbar region when dissecting the conjunctival autograft, or from the same region from patients who underwent extracapsular cataract extraction.

### Tear sample collection

Patients were evaluated with a slit lamp to exclude any disease of the anterior segment. Tear samples were collected using a 5-μl calibrated glass micro capillary tube (Blauband intra MARK, Brand GMBH, Werthein, Germany) without touching the eye or eyelids. Samples were taken at different times of the day to avoid diurnal variations [11]. One sample typically contained 50 μl. After collection, samples were centrifuged at 14,000 × g for 1 min at 4°C to remove cellular debris and stored at −20°C until required.

### Tissue processing

#### Histology and immunohistochemistry

Pterygia and normal conjunctivas were fixed in 10% formaldehyde. Sections were routinely stained with hematoxylin and eosin [12]. Each section (5-μm thickness) was incubated with the appropriate primary antibody for 24 h in a humidified chamber at 4°C. The primary antibody was polyclonal rabbit anti-HCC (diluted 1:100; Ab7653, Abcam). Antibody was diluted in phosphate-buffered saline (PBS) containing 0.2% (v/v) Triton X-100 and 3% (w/v) bovine serum albumin (BSA). Sections were washed, and then incubated with goat anti-rabbit conjugated to horseradish peroxidase enzyme (1:100; AP132, Serotec, AP132) in a humidified chamber for 2 h at room temperature. Sections were developed with diaminobenzidine (Sigma) until brownish staining was evident.

#### Digital morphometry

**Figure 1:**
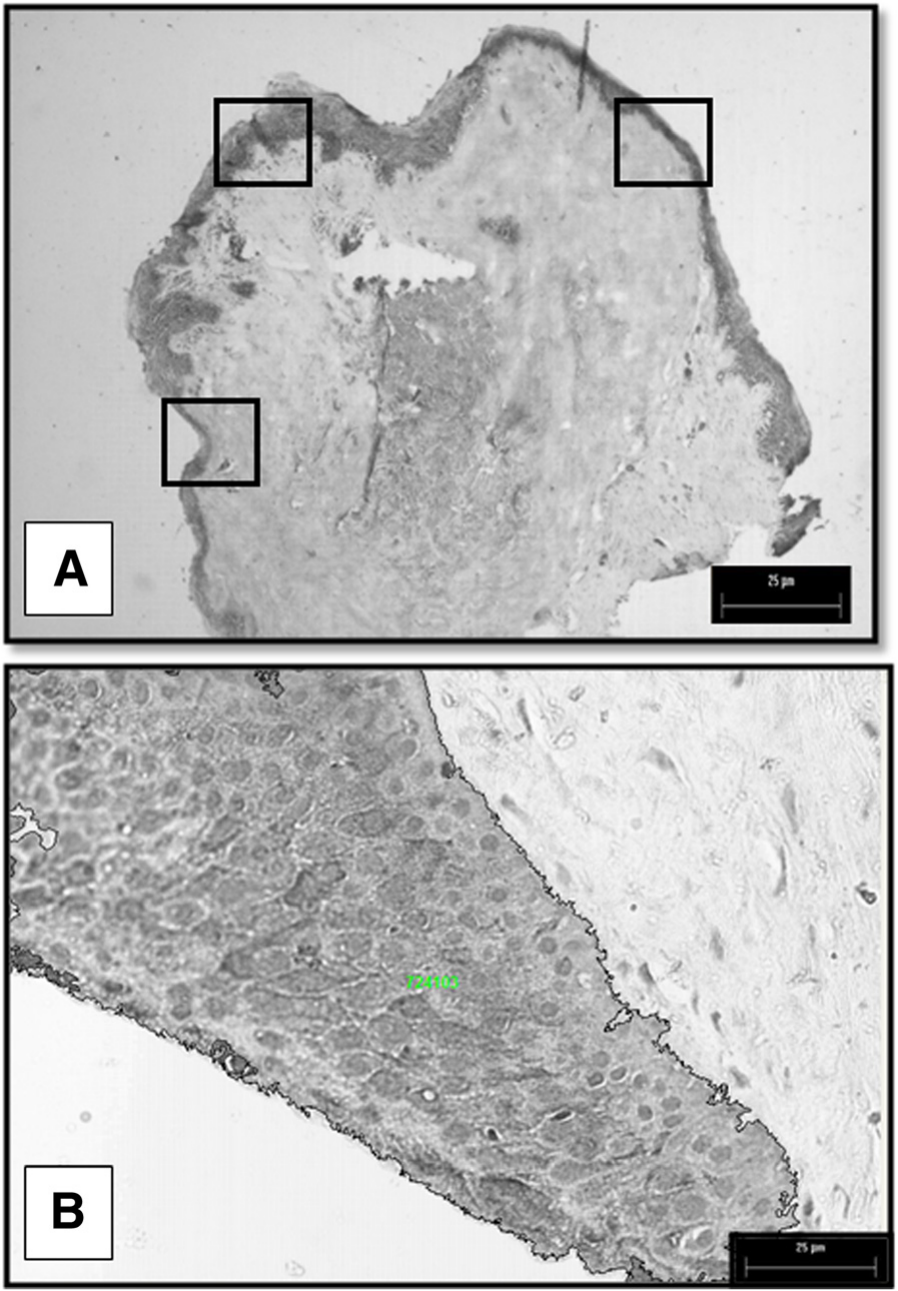
**(A) Morphological approach used to select the optimal field of view to quantify HCC in surgical tissue samples; the continuous lines' squares represent epithelial fields quantified.** (**B**) Example of an area of pterygium chosen for the HCC quantifying.

We used an Axioscop 40 (Carl Zeiss) light microscope equipped with a digital image acquisition system. For each sample examined, 10 randomly chosen fields from epithelial tissue represented by continuous lines squares in Figure 1A were analyzed. HCC area was defined as the ratio of an epithelium per unit of tissue as showed in Figure 1B. Image Pro Plus software (Media Cybernetics, Silver Spring, MD) was used to measure epithelial areas [13,14].

#### RNA extraction and cDNA synthesis

RNA was extracted from individual pterygium samples and pooled normal conjunctivas. RNA was extracted from 5 mg of tissue using a total RNA Isolation System (Promega, Madison, WI) according to the manufacturer’s instructions. Purity and quality of RNA were assessed with the Nanodrop 1000 system (Thermo Scientific, Wilmington, DE). We obtained 90 ± 10 ng of RNA for each pterygium, and 100 ± 10 ng of RNA for pooled conjunctiva samples. Synthesis of cDNA was conducted using SuperScript™ first strand synthesis for RT-PCR (Invitrogen, Groningen, The Netherlands) at 55°C for 45 min. Primers for the PCR were designed using DNAstar 4.02 software. Primer sequences were 5^′^-GCG GCG TGC ACT GGA CTT TG-3^′^ (HCC primer forward) and 5^′^-GCC GCC TGC TGC CTT CTC TG-3^′^ (HCC primer reverse). The PCR consisted of 1 μl of cDNA, 1.5 mM MgCl_2_, 0.2 mM dNTP, 0.5 μM each primer and 2.5 U of Taq DNA polymerase. Thermal cycling involved a denaturing step at 94°C for 5 min, followed by 25 cycles of denaturation (94°C, 5 min) and annealing (61°C, 1 min), then a final extension step at 72°C for 2 min. All reactions were performed in duplicate. Amplicons were visualized by electrophoresis on 1.5% (w/v) agarose gels stained with ethidium bromide.

#### ELISA

Of the 74 tear samples we analyzed, 28 were from patients with primary pterygium; 22 were from recurrent pterygium, and 24 were from a normal conjunctiva. In the wells of a 96-well plate, 50 μl of a tear sample was added to 50 μl of carbonate/bicarbonate buffer (pH 10) to coat each well. Plates were incubated at 4°C overnight. We added 0.1% (w/v) BSA in PBS (pH 7.4) to each well for 45 min at 37°C, then performed three 5-min washes with PBS containing 0.05% (v/v) Tween-20 (PBS-Tw). Rabbit anti-HCC (diluted 1:1000 in PBS-Tw) was added to wells and incubated for 1.5 h at 37°C. Wells were washed with PBS-Tw, and then incubated with goat anti-rabbit IgG conjugated to horseradish peroxidase (diluted 1:2500 in PBS-Tw) for 1 h at room temperature. Wells were again washed with PBS-Tw, and ortho phenylenediamine added for color development. Absorbance values for each well were determined at 490 nm in a iMark (Bio Rad) spectrophotometer [15].

#### Protein extraction

Frozen tissue sections from pterygium and conjunctiva were placed in a glass tissue macerator (Kontes 15 ml) with lysis buffer (10 mM Tris–HCl pH 7.4, 50 mM NaCl, 3 mM iodoacetamide, three Mm (3S)-1-chloro-3-tosylamido-7-amino-2-heptanone hydrochloride TLCK and 1 mM phenylmethylsulfonyl fluoride PMSF). The proportion of tissue to lysis buffer was 1 g/ml. Tissues were lysed with 100 strokes, and samples kept on ice; however, it was necessary to centrifuge samples at 15,000 rpm for 10 min at 4°C as they became dense. We obtained two phases: the bottom phase corresponded to membrane contents, and the supernatant contained the contents of the cytosol. These phases were stored at −20°C until required. We subjected human serum to an LC10 column (Beckman Coulter Inc., Fullerton, CA) according to the manufacturer’s instructions. Briefly, 100 μL of serum was diluted 5-fold with buffer A and injected onto the column in buffer A at a flow rate of 0.5 ml/min for 25 min, then 2.0 ml/min for 5 min on a Shimadzu LC10A VP system (Shimadzu Co., Kyoto, Japan). After collection of the flow-through fraction containing unbound proteins, the column was washed and bound proteins were eluted with buffer B (stripping buffer) at a flow rate of 2.0 ml/min for 18 min. Fractions were collected into 1.5 ml micro centrifuge tubes.

#### Quantitation of proteins

A standard curve was constructed using BSA (range 0–1 mg/ml). Samples (1 μl of membrane or cytosol contents) were added to 1 ml of Bradford's solution [16]. The absorbance of samples was determined at 595 nm.

#### Immunoblot analysis

A positive control was taken from the serum patients with chronic renal failure. Pterygia and conjunctiva tissues containing 100 μg of protein were mixed with an equal volume of buffer and then boiled for 5 min. Samples from human serum were dissolved in 15 μl of sample preparation buffer, mixed well, and subjected to sodium dodecyl sulfate (SDS) polyacrylamide gel electrophoresis according to Laemmli protocol [17]. Samples were loaded onto a 7.5% separating gel, electrophoresed and transferred to a nitrocellulose membrane. Membranes were cut into strips and immersed in a blocking reagent (10% skim milk in 50 mM Tris–HCl pH 7.4 and 200 mM NaCl). The blocking reagent was removed and rabbit anti-HCC (diluted 1:1000 in blocking buffer) was added and incubated for 3 h at 37°C. After washing, peroxidase-conjugated anti-rabbit IgG antibody (diluted 1:1000 in blocking buffer) was added and incubated for 2 h at 37°C. Reactions were developed using a substrate solution (H2O2 containing 3.6 mM 4-chloro-1-naphthol).

## Results

### RT-PCR detection of HCC

**Figure 2:**
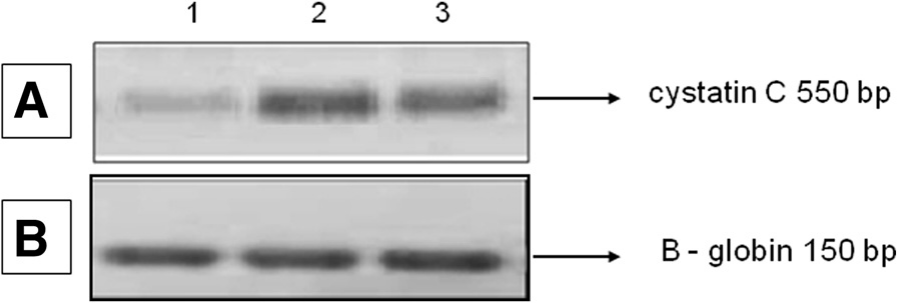
**(A) Using primers specific for HCC, a 550 bp amplicon was amplified in conjunctiva, active recurrent pterygium, and active primary pterygium (lanes 1, 2 and 3 respectively) samples.** (**B**) A portion of the constitutive B-globin gene was amplified by RT-PCR (150 bp) in conjunctiva, active recurrent pterygium, and active primary pterygium (lanes 1, 2 and 3 respectively) samples.

Differential expression of HCC was observed, with an over expression in recurrent and primary pterygia compared with a normal conjunctiva (Figure 2B).

### Morphological characteristics of conjunctiva and pterygium

**Figure 3:**
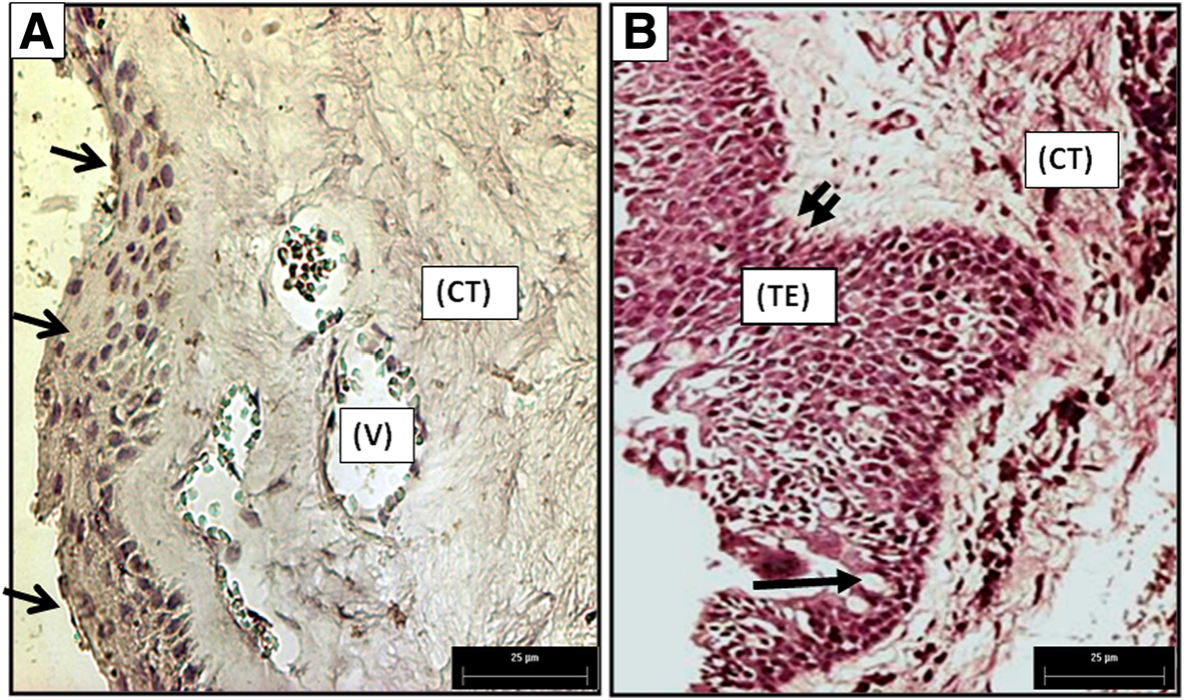
**(A) A healthy conjunctiva lacking evidence of inflammatory cell infiltration, containing few vessels (V), normal epithelium (arrows), and uniform connective tissue (CT).** (**B**) Primary Pterygium. Thickened epithelium (TE), epithelial goblet cells (arrow), and basal lamina (double arrow), on its right, we identified connective tissue (CT) (400× Magnification).

In the healthy conjunctiva, we observed non-keratinized stratified squamous epithelium with four to eight layers of cells (Figure 3A). These tissues had a well-defined basement membrane that separated the underlying connective tissue (CT) containing blood vessels (V) and other normal cells of the connective tissue. Inflammatory infiltrates were absent from normal tissue, and they contained an orderly arrangement of collagen fibers (Figure 3A). This contrasts with the pterygia tissue samples which had thickened epithelial areas of 10 or more layers (TE in Figure 3B), goblet cells (arrow in Figure 3B) concentrated in the crypts of Henle, and a well-defined basal lamina (double arrow in Figure 3B). The underlying connective tissue (CT) presents an increased number of cells, and other areas were characterized by increased density of collagen. Additionally, in areas close to blood vessels, infiltrates were rarely observed (Figure 3B).

### HCC immunohistochemistry

**Figure 4:**
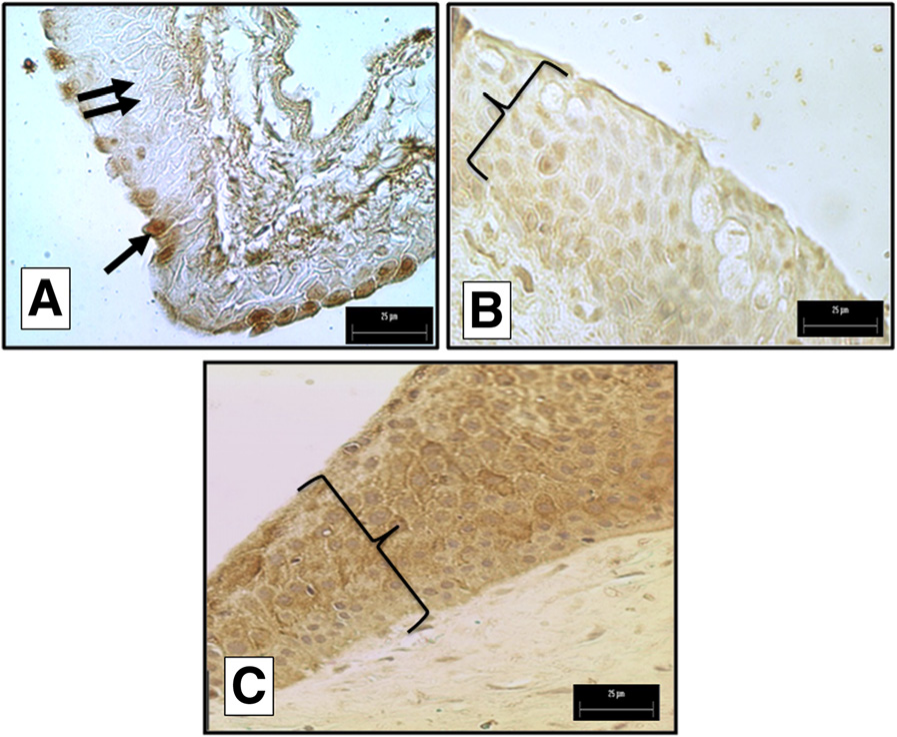
**(A) Superficial epithelial cells in the healthy conjunctiva were positive for the presence of HCC (arrow).** (**B**) Most of the epithelial tissue was positive for HCC in primary pterygium (key). (**C**) Nearly all the epithelial tissue was positive for HCC in recurrent pterygium (key; 400× Magnification).

In the normal conjunctiva, we detected HCC in most apical epithelial cells (arrow in Figure 4A) meantime, other epithelial cells were negative for presence of HCC (double arrow in Figure 4A). In primary pterygium (Figure 4B), we observed HCC faintly in all areas of the thickened epithelium (key in Figure 4B) with homogeneous staining across layers, from basal to apical cells. We detected solid expression of HCC in the epithelium of recurrent pterygium (key in Figure 4C) in cells from the basal, middle and apical layers, and we could quantify by morphometry (Figure 1A). HCC was not observed in CT cells.

### Digital morphometric analysis

**Figure 5:**
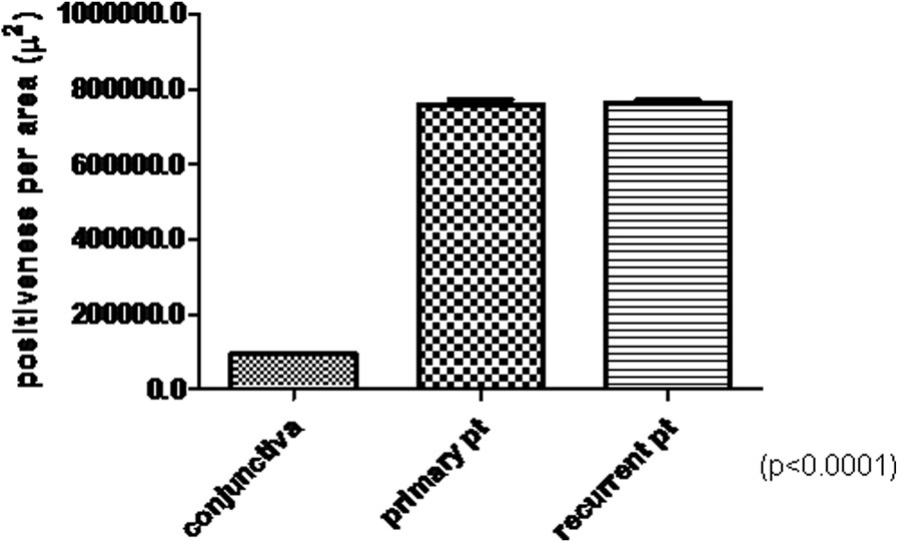
**Quantitation of reactivity to HCC antibody in conjunctivas, primary pterygia and recurrent pterygia.** HCC levels in conjunctivas were lower compared with those in primary and recurrent pterygia (p < 0.0001). The area of reactivity between primary and recurrent pterygia was not significantly different.

From the images that we obtained we saw that samples in the pterygia groups contained larger positive areas in terms of distribution regarding the conjunctiva (Figure 5). We compared data sets for three cases using an unpaired Student’s t-test. The mean of the area in the primary pterygia and the conjunctiva showed significant differences between groups (*p* < 0.0001). A comparison of recurrent pterygia and normal conjunctivas, or of primary and recurrent pterygia, indicated no significance.

### Western immunoblot detection of HCC

**Figure 6:**
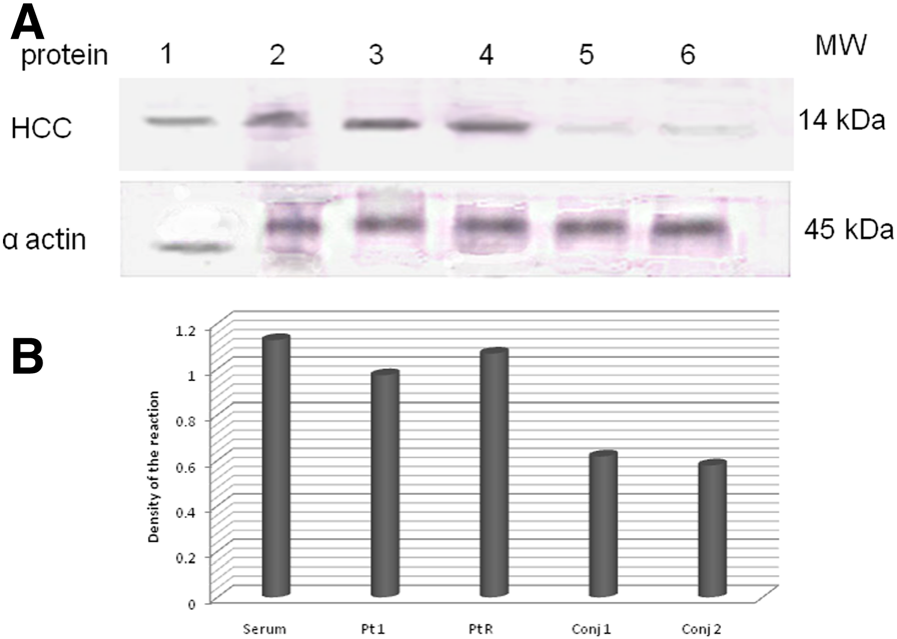
**Detection of HCC. A**) Immunoblot. Lane 1, molecular weight markers; 2, serum of chronic renal failure sample (positive control); 3, primary pterygium; 4, recurrent pterygium; 5 and 6, healthy conjunctiva. Molecular Weight Human Cystatin C 17 kDa; α actin 45 kDa. **B**) Density analysis of immuno localized protein HCC in Western blot, we observed height density in Recurrent pterygium as in positive control.

HCC expression levels were increased in active primary and active recurrent pterygia (Figure 6) as determined by western immunoblotting. Expression levels of HCC were lower in the conjunctiva compared with the pterygia samples (Figure 6A). The protein that we detected corresponded to a 14 kDa, as described in the literature. Taken into consideration of the house keeping alfa actin expression, we detected that the presence of HCC in recurrent pterygium was highest as to the positive control, secondly was primary pterygium, finally the expression of this protein was weakly in conjunctive samples (Figure 6B).

### ELISA detection of HCC in tear samples

**Figure 7:**
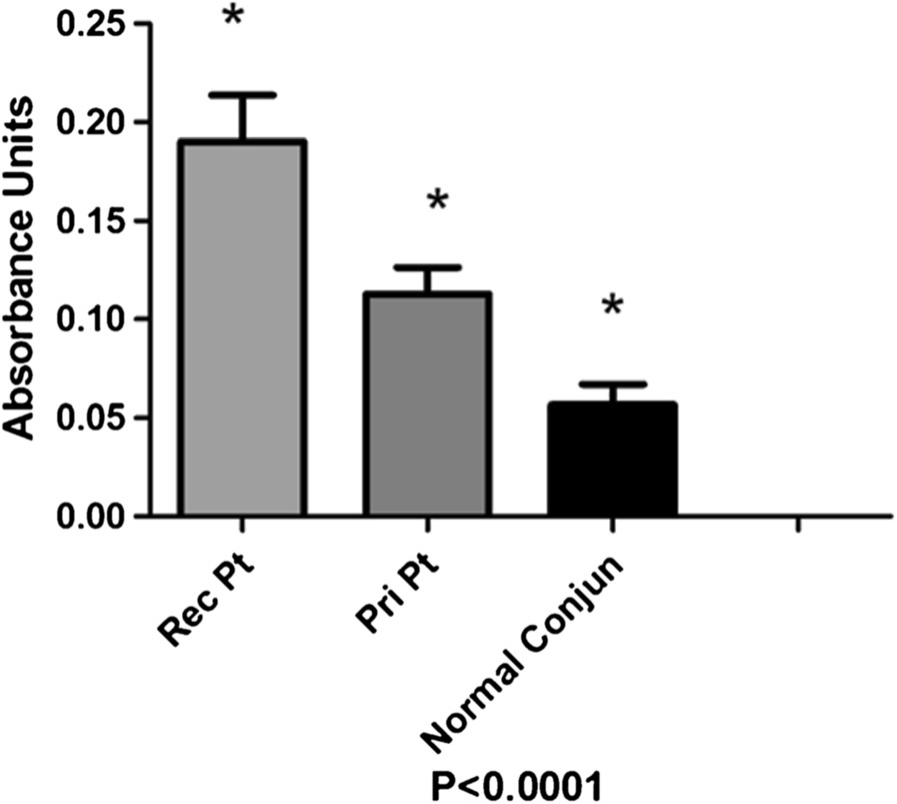
**Quantitation of HCC by ELISA in tears from active recurrent pterygium, active primary pterygium and healthy individuals.** Absorbance values were increased in recurrent pterygia compared with primary pterygia. The amount of HCC in the tears of patients with active recurrent pterygium was 33% higher than that in healthy subjects.

Our analysis showed that HCC molecules were present in the tear film of all patients and controls. In healthy people, the concentration of HCC was lower (0.05 UA ± 0.0003) than in patients with pterygium (0.13 UA ± 0.02; Figure 7); and this difference was statistically significant (P < 0.0002). We saw a higher concentration of HCC in the tear film of patients with recurrent pterygium (0.19 UA ± 0.03) compared to patients who had primary pterygium (0.10 UA; P < 0.0003). Sub-analysis of patients with pterygium showed that patients with the active disease had higher concentrations of HCC than those with inactive disease, for either the primary (0.12 vs. 0.08 UA) or recurrent (0.18 vs. 0.15) scenarios (P < 0.04; Figure 7 and Table 1).

**Table 1:** Comparison of Tukey’s multiple statistical samples for tears from active primary and recurrent pterygium, and healthy conjunctiva

**Tukey’s multiple comparison test**	**Mean diff.**	**q**	**Significant?**	**Summary**	**95% CI of diff**
Rec PtAct vs Pri Pt Act	0.07755	14.77	Yes	***	0.05875 to 0.09634
Rec PtAct vs Normal Conjun	0.1338	25.48	Yes	***	0.1150 to 0.1526
Pri Pt Act vs Normal Conjun	0.05627	10.71	Yes	***	0.03748 to 0.07506

## Discussion

Our study involved tissue samples (pterygium and conjunctiva) obtained from surgery, and tears from patients, with and without pterygium. All patients who underwent surgery had active pterygium. Conversely, outpatients with pterygium were classified as active if they presented with vascularization, edema and active hyperaemia, or as inactive if they showed a fibrotic component. Using RT-PCR we found that HCC was expressed in both normal conjunctiva and pterygium, supporting the findings of Chang et al., who found HCC in another mucosa [11]. Abrahamson et al., found HCC in several autopsy tissue specimens (heart, kidney, lung, liver, uterus and seminal vesicles) and in several fluids (tears, saliva, cerebrospinal fluid and seminal plasma) [18]. Wasselius et al., studied cystatin C in the anterior segment of rat and mouse eyes and found that expression of the protein was localized to the ciliary body, cornea and retina [19]. In that study, cystatin C was not detected in rat and mouse conjunctiva. Our results presented here contradict those presented by Wasselius et al.; this may be because of the anatomical and histological variations between rodent and human eyes. Other authors have noted that HCC plays an important role in the regulation of proteolysis and inflammatory diseases, controlling the activity of cathepsin B in tissues and fluids, including tears [20].

Using immunohistochemistry, we found that normal conjunctiva contained HCC in the most superficial epithelial layers. In contrast, HCC was expressed in all epithelial tissues of pterygia, from basal epithelial cells to the most superficial layer of the epithelium. It is interesting to note that this compartment has a high proliferatives rate in comparison with a normal conjunctiva, such as morphological changes we observed in a picture from two tissues stained with H&E technique (Figures 3A and B). Other authors have studied HCC by immunohistochemistry but in different tissues. Chang et al., showed light immunostaining intensity corresponding to the presence of HCC in normal sinus mucosa, and intense staining in an inflamed sinus mucosa [11]. Based on the anti-inflammatory function(s) of HCC, Chang et al., suggest that this protein may play an important role in the protection of normal sinus mucosa, and also in the prevention of aggravation of inflammatory conditions that occur in chronic sinusitis [11]. Hung et al., compared cystatin C expression in normal human oral mucosa and oral submucous fibrosis (OSF). He demonstrated that OSF was expressed at levels significantly higher than HCC in normal oral mucosa at the RNA and protein level [21]. They hypothesized that cystatin C had synergistic effects with other antimicrobial substances such as lysozyme, lactoferrin, and defensins, that make it more potent against infectious pathogens. It contrast, it has been shown that the balance between cystatin C and C1 cathepsin is of major importance in the regulation of proteolytic activity under normal physiological conditions, and for pathological degradation that occurs in inflammatory diseases. Our findings may be explained in the context of the protective role(s) of HCC; the protein may be trying to maintain homeostatic redox at the anterior surface of the eye.

Our ELISA and western blot results indicate low levels of HCC in the tears and tissues of patients with a normal conjunctiva. The amount and concentration of HCC were increased in the tears and tissues from patients with pterygium, with the highest levels observed in patients with active recurrent pterygium. Sharman et al., assess the concentration of HCC in gingival crevicular fluid (GCF) and serum from patients with various periodontal diseases, the mean HCC concentration in GCF and serum was observed to be highest in patients with periodontitis, and lowest in healthy individuals, they suggested that HCC levels increased with disease progression to prevent further periodontal degeneration; HCC levels then decrease after treatment to maintain bone metabolic homeostasis [22]. Lertnawapan et al., found that concentrations of cystatin C were significantly higher in the sera of patients with Systemic Lupus Erythematosus (SLE) compared with controls. These increased cystatin C levels correlated with increases in erythrocyte sedimentation rate, Tumor Necrosis Factor-alpha, and Interleukin 6, suggesting that cystatin C concentrations in SLE may be affected by inflammatory mechanisms [23]. Henskens et al., found that levels of cystatin C were increased in the saliva of patients with inflammatory periodontal disease in comparison with healthy people [24]. Consider these findings and combined with our results presented here, point to a possible role for HCC in fluids, as some sort of acute-phase protein during inflammatory diseases.

We found that HCC was present to a great extent in epithelial cells of pterygium samples. Underlying cells in CT did not exhibit strong staining for HCC, suggesting that the production of HCC is confined to epithelial cells. This further indicates that pterygium does not involve a clear inflammatory process. Based on these findings, we can deduce that HCC acts as an epithelial proliferatives stimulant, and at the same time as a modulator of inflammation in underlying CT. This is similar to what was outlined by Koroloenko et al., (2008) [25] and Tavera et al., (1990) [26].

## Conclusion

Our study has established that HCC is expressed in normal conjunctiva and that this protein is up regulated in proliferatives diseases of the ocular surface, such as pterygium. This suggests that HCC may play a key role(s) in the protection of a conjunctiva against aggressors, and may regulate the inflammatory ocular response. The molecular mechanism and potential of HCC as a prognostic biomarker for activity or recurrence of pterygium remains an area that requires further investigation.

## Abbreviations

HCC: Human cystatin C; RT-PCR: Reverse-transcription polymerase chain reaction; ELISA: Enzyme-linked immunosorbent assay; ECM: Extracellular matrix; MMP: Matrix metalloproteinase; kDa: Kilodaltons; PBS: Phosphate-buffered saline; BSA: Bovine serum albumin; RNA: Ribonucleic acid; DNA: Deoxyribonucleic acid; PCR: Polymerase chain reaction; GCF: Gingival crevicular fluid; SLE: Systemic lupus erythematosus; ESR: Erythrocyte sedimentation rate; TNF-α: Tumor necrosis factor-alpha; IL-6: Interleukin 6; Pri: Primary; Rec: Recurrent; Pt: Pterygia.

## Competing interests

The authors declare that they have no financial interest in any of the products used in this research.

## Authors’ contributions

BGLF carried out the molecular genetic and immunohistochemistry studies, participated in the design of the study, and drafted the manuscript. VJJ carried out the immunohistochemistry studies, participated in data analysis and helped draft the manuscript. KD critically revised the manuscript. GCR carried out molecular genetic studies and participated in the design of the study. TBE carried out the immunoassays and their statistical analysis. TBLF conceived of the study, helped design and coordinate experiments, and helped to draft the manuscript. All authors read and approved the final manuscript.

## Pre-publication history

The pre-publication history for this paper can be accessed here:

http://www.biomedcentral.com/1471-2415/13/6/prepub
